# Draft Genome Sequences of 14 *Lactobacillus*, *Enterococcus*, and *Staphylococcus* Isolates from the Nasopharynx of Healthy Feedlot Cattle

**DOI:** 10.1128/MRA.00534-19

**Published:** 2019-08-22

**Authors:** Samat Amat, Devin B. Holman, Edouard Timsit, Katherine E. Gzyl, Trevor W. Alexander

**Affiliations:** a Lethbridge Research and Development Centre, Agriculture and Agri-Food Canada, Lethbridge, AB, Canada; b Department of Production Animal Health, Faculty of Veterinary Medicine, University of Calgary, Calgary, AB, Canada; c Lacombe Research and Development Centre, Agriculture and Agri-Food Canada, Lacombe, AB, Canada; University of Maryland School of Medicine

## Abstract

Here, we present the first draft genome sequences of 14 bacterial strains isolated from the nasopharynx of healthy feedlot cattle. These genomes are from 12 *Lactobacillus* isolates (L. amylovorus, L. buchneri, L. curvatus, and L. paracasei), 1 Enterococcus hirae isolate, and 1 Staphylococcus chromogenes isolate.

## ANNOUNCEMENT

The nasopharynx of feedlot cattle is inhabited by a rich and diverse microbial community (1). Within the nasopharynx, opportunistic bacterial pathogens involved in bovine respiratory disease (BRD), also known as shipping fever, are also present as part of the nasopharyngeal (NP) microbiota of healthy cattle. When cattle experience compromised immunity due to stress and viral infection, these respiratory pathogens can proliferate in the nasopharynx and translocate into the lung, where they can cause bronchopneumonia (2). Recent studies have suggested that certain members of the NP microbiota have an important role in maintaining respiratory health in feedlot cattle by providing resistance against colonization by BRD-associated pathogens (3, 4). Therefore, we recently isolated commensal bacteria from the nasopharynx of healthy feedlot cattle that may have potential for inhibition of bovine respiratory pathogens such as Mannheimia haemolytica. We selected 14 of these isolates for whole-genome sequencing.

All bacteria were isolated using deep nasopharyngeal swabs from the nasopharynx of healthy feedlot cattle and were taxonomically identified by sequencing the nearly full-length bacterial 16S rRNA gene (>1,400 bp) as described previously (5). Biochemical identification was also performed on these isolates. In brief, the isolates were subcultured on *Lactobacillus* De Man, Rogosa, and Sharpe (MRS) agar (Dalynn Biologicals, Calgary, AB, Canada), and colony morphologies were observed after 24 to 48 h at 39°C. Anaerobic growth was also assessed on MRS agar or tryptic soy agar at 39°C in an anaerobic chamber with an atmosphere of 85% nitrogen, 10% hydrogen, and 5% CO_2_. Acid production from carbohydrates was determined with the API 50 CHL gallery (bioMérieux, Saint-Laurent, QC, Canada; *Lactobacillus* and *Enterococcus*) per the manufacturer’s instructions. Confirmatory identifications were obtained through comparison with published results.

Each isolate was grown in Difco Lactobacilli MRS broth (BD, Mississauga, ON, Canada) at 37°C for 18 h and centrifuged at 13,000 × *g* for 5 min, and genomic DNA was extracted from the pellet using a DNeasy tissue kit (Qiagen, Inc., Mississauga, ON, Canada) as described previously (5). The concentration and quality of extracted genomic DNA were measured using a NanoDrop 2000 spectrometer (Thermo Fisher Scientific, Wilmington, DE, USA). Subsequently, the extracted DNA was purified and concentrated using the Genomic DNA Clean & Concentrator kit (Zymo Research, Irvine, CA, USA). Genomic libraries were prepared using a Nextera XT DNA library prep kit (Illumina, Inc., San Diego, CA, USA) and sequenced on an Illumina NextSeq 500 instrument with the 500/550 midoutput 300-cycle kit following the manufacturer’s instructions. Pre- and postprocessed reads were assessed for quality using FastQC v.0.11.1 (6). Trimmomatic v.0.38 (7) was used to remove sequencing adapters, reads with a quality score of less than 15 over a sliding window of 4 bp, and sequences shorter than 50 bp. The leading and trailing 15 bp were also removed from each sequence. Reads were assembled using SPAdes v.3.11.1 (8) with the default parameters in the “careful” mode, and the quality of the assemblies was determined using QUAST v.5.0.1 (9). The taxonomy of the assemblies was confirmed using Kraken 2 v.2.0.7beta and the minikraken2 database v.2 with the default parameters (10). Assemblies were then annotated using Prokka v.1.13.3 with the default parameters and a minimum contig length of 500 bp (11). The assembly statistics and number of coding sequences for each assembled genome are shown in Table 1. The draft genomes of these 14 isolates will be further characterized to evaluate encoded mechanisms that may lead to inhibition of the BRD pathogen *M. haemolytica*.

**TABLE 1:** Bovine nasopharyngeal *Lactobacillus*, *Enterococcus*, and *Staphylococcus* sp. assembly statistics with various read coverages

BioSample accession no.	Sample ID^*a*^	Strain ID^*a*^	Species	Genome assembly no.	SRA accession no.	No. of contigs	No. of reads	Genome size (bp)	*N*_50_ value (bp)	Avg coverage (×)	No. of coding sequences	G+C content (%)
SAMN11456257	64C	S44	*E. hirae*	GCA_005047985	SRX5705612	58	1,174,501	2,782,597	121,368	63	2,523	38
SAMN11456246	72B	S60	*L. amylovorus*	GCA_005049155	SRX5705619	74	1,341,569	2,004,240	48,176	100	2,014	37.9
SAMN11456247	65E	S43	*L. buchneri*	GCA_005049145	SRX5705609	40	1,204,517	2,498,525	235,856	72	2,361	44.3
SAMN11456248	38C	S45	*L. buchneri*	GCA_005047285	SRX5705611	25	1,327,600	2,493,955	610,029	80	2,423	44.2
SAMN11456249	86A	S47	*L. buchneri*	GCA_005048055	SRX5705613	27	947,772	2,445,621	245,952	58	2,304	44.4
SAMN11456250	65A	S50	*L. buchneri*	GCA_005047235	SRX5705608	44	1,040,870	2,544,838	221,549	61	2,418	44.1
SAMN11456251	86D	S51	*L. buchneri*	GCA_005049245	SRX5705607	90	1,186,391	2,535,187	65,294	70	2,441	44.2
SAMN11456253	67A	S59	*L. buchneri*	GCA_005049205	SRX5705620	37	1,491,108	2,542,267	245,952	88	2,417	44.1
SAMN11456256	65B	S58	*L. buchneri*	GCA_005047575	SRX5705617	38	1,686,689	2,505,127	245,977	101	2,360	44.3
SAMN11456252	63A	S53	*L. buchneri*	GCA_005047265	SRX5705618	38	1,672,438	2,498,046	245,952	100	2,362	44.3
SAMN11456255	65G	S42	*L. buchneri*	GCA_005048025	SRX5705610	38	996,918	2,497,693	245,952	60	2,359	44.3
SAMN11456254	103C	S46	*L. curvatus*	GCA_005049195	SRX5705614	67	1,526,957	1,871,416	66,928	122	1,857	41.9
SAMN11456259	3E	S49	*L. paracasei*	GCA_005049135	SRX5705615	92	1,375,140	3,016,142	86,730	68	2,888	46.2
SAMN11456258	28C	S48	*S. chromogenes*	GCA_005048075	SRX5705616	37	1,224,373	2,392,851	238,895	77	2,335	36.6

a ID, identification.

### Data availability.

All raw genome sequences and draft genome assemblies have been deposited in the Sequence Read Archive and GenBank, respectively, under the accession numbers listed in Table 1.
